# Remote monitoring of *Cydia pomonella* adults among an assemblage of nontargets in sex pheromone‐kairomone‐baited smart traps

**DOI:** 10.1002/ps.6433

**Published:** 2021-05-14

**Authors:** Michele Preti, Riccardo Favaro, Alan Lee Knight, Sergio Angeli

**Affiliations:** ^1^ Faculty of Science and Technology Free University of Bozen‐Bolzano Bolzano Italy; ^2^ Instar Biologicals Yakima WA USA

**Keywords:** codling moth, Tortricidae, Sesiidae, automatic detection, electronic trap, pome fruit

## Abstract

**BACKGROUND:**

Captures of codling moth, *Cydia pomonella* (L.), in traps are used to establish action thresholds and time insecticide sprays. The need for frequent trap inspections in often remote orchards has created a niche for remote sensing smart traps. A smart trap baited with a five‐component pheromone‐kairomone blend was evaluated for codling moth monitoring among an assemblage of other nontargets in apple and pear orchards.

**RESULTS:**

Codling moth captures did not differ between the smart trap and a standard trap when both were checked manually. However, the correlation between automatic and manual counts of codling moth in the smart traps was low, *R* ^2^ = 0.66 ÷ 0.87. False‐negative identifications by the smart trap were infrequent <5%, but false‐positive identifications accounted for up to 67% of the count. These errors were primarily due to the misidentification of three moth species of fairly similar‐size to codling moth: apple clearwing moth *Synanthedon myopaeformis* (Borkhausen), oriental fruit moth *Grapholita molesta* (Busck), and carnation tortrix *Cacoecimorpha pronubana* (Hübner). Other false‐positive counts were less frequent and included the misidentifications of dipterans, other arthropods, patches of moth scales, and the double counting of some moths.

**CONCLUSION:**

Codling moth was successfully monitored remotely with a smart trap baited with a nonselective sex pheromone‐kairomone lure, but automatic counts were inflated in some orchards due to mischaracterizations of primarily similar‐sized nontarget moths. Improved image‐identification algorithms are needed for smart traps baited with less‐selective lures and with lure sets targeting multiple species.

## Graphical abstract

*Cydia pomonella* monitoring with smart traps baited with pheromone‐kairomone lures enabled remote detection of the target species, but also of other pests, including *Grapholita molesta*, *Synanthedon myopaeformis*, and *Cacoecimorpha pronubana*.

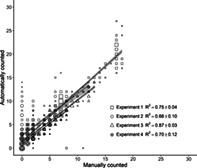

© 2021 The Authors. *Pest Management Science* published by John Wiley & Sons Ltd on behalf of Society of Chemical Industry.

## 1 INTRODUCTION

Action thresholds based on moth captures in traps are widely used in integrated pest management programs in pome fruit to aid the management of codling moth, *Cydia pomonella* (L.) (Lepidoptera: Tortricidae).^1^ Growers in many production regions rely on traps baited with sex pheromone ((*E,E*)‐8,10‐dodecadien‐1‐ol, codlemone) for monitoring, and orchards are treated with sex pheromone dispensers for mating disruption (MD).^2^, ^3^, ^4^, ^5^ Unfortunately, these practices can have three important limitations. First, sex pheromone‐baited traps capture only males and provide at best only an indirect measure of egg laying and larval eclosion timing.^6^ Second, the reliability of sex pheromone‐baited traps in MD‐treated orchards to accurately predict these later biological events can be low if the traps inhibited by MD fail to capture more than an occasional moth.^7^ Third, monitoring programs can be expensive, and time‐sensitive recommendations can be delayed in a management program with field visits to check traps typically scheduled once per week.^8^

Fortunately, during the last few decades significant progress has been achieved in monitoring codling moth in MD‐treated orchards, including the use of (*E,Z*)‐2,4‐ethyl decadienoate (pear ester) alone and in combination with codlemone, acetic acid, and plant volatiles.^9^, ^10^, ^11^, ^12^ Most recently, a five‐component sex pheromone‐kairomone blend of codlemone, pear ester, (*E*)‐4,8‐dimethyl‐1,3,7‐nonatriene (DMNT), 6‐ethenyl‐2,2,6‐trimethyloxan‐3‐ol (linalool oxide pyranoid), and acetic acid has increased the captures of codling moths compared with earlier blends, and even outperformed sex pheromone lures in both MD‐treated and untreated orchards.^13^, ^14^

Other advances in pest monitoring in pome fruits have included the development of automatic pest detection devices, here referred to as smart traps, equipped with cameras, connected via the internet, and running pest identification software.^15^, ^16^ Several smart traps with different levels of automatization have been developed and are commercially available, e.g. Trapview traps, iSCOUT traps, SightTrap traps, Z‐Trap 1, and DTN Smart Trap traps.^17^, ^18^, ^19^, ^20^ Similarly, a number of smart trap prototypes have been investigated for monitoring tortricid orchard pests, including codling moth, oriental fruit moth, *Grapholita molesta* (Busck), and European grapevine moth, *Lobesia botrana* (Denis and Schiffermüller) (Lepidoptera: Tortricidae).^21^, ^22^, ^23^ However, these experimental studies have only used traps baited with sex pheromone lures and captures of nontargets in these traps has not been reported to be a serious issue. Thus, there is limited information to judge how reliable smart trap performance would be if baited with less selective lures and having to sort a more complex array of images.

The new five‐component sex pheromone‐kairomone blend reported in Knight *et al*.^13^, ^14^ comprises several volatiles which can be attractive to other insect pests besides codling moth. For example, acetic acid alone or in combination with host plant volatiles can be attractive for many noctuid moths^24^, ^25^ and tortricid pest species, including obliquebanded leafroller, *Choristoneura rosaceana* (Harris), apple pandemis, *Pandemis pyrusana* (Kearfott), and eye‐spotted budmoth, *Spilonota ocellana* (Denis and Schiffermüller) (Lepidoptera: Tortricidae).^26^, ^27^, ^28^, ^29^ Pear ester can be attractive for tortricid pests of chestnut (*Castanea sativa* Mill.) species, such as *Cydia fagiglandana* (Zeller), *Cydia splendana* (Hübner), and *Pammene fasciana* (L.), and green budmoth, *Hedya nubiferana* (Haworth) (Lepidoptera: Tortricidae).^30^ Pear ester has also been reported to be attractive for the oriental fruit moth.^31^ A blend of pear ester and acetic acid has been shown to be attractive for the apple clearwing moth, *Synanthedon myopaeformis* (Borkhausen) (Lepidoptera: Sesiidae).^32^ Thus, the use of this new and more effective lure for codling moth in smart traps may require more acute visual differentiation of images to correctly count one or more pest species within the same trap. At present, it is not clear if two co‐occurring pome fruit pests with slight differences in body size can be reliably differentiated by smart traps, e.g. mean body length of codling moth, 86 mm versus 66 mm for oriental fruit moth.^33^

Our study selected one type of commercial smart trap, Trapview, for this evaluation using the new five‐component lure for codling moth across a range of orchards in the Emilia‐Romagna region of Italy. First, we assessed the smart trap's performance to capture codling moth compared with a standard delta‐shaped trap due to physical differences in their construction and in the type of adhesive used on the trap liners. Then, studies were established in orchards with various densities of several nontarget species, including other important pests, to determine the occurrence of both false‐positive counts (miscounting other insects as codling moth) and false‐negative counts (missing counts of codling moth).

## 2 MATERIAL AND METHODS

### 2.1 Description of traps and lures

Two trap types were deployed in this study. The first was an orange delta‐shaped trap (ODT) (Pherocon VI; Trécé Inc. Adair, OK, USA). The ODT was 27 cm long × 20 cm wide × 11 cm tall with two 56‐cm^2^ triangular openings and used a removable sticky liner (289 cm^2^) coated with a polybutene adhesive. The second was a camera‐equipped green smart trap (GST) (Trapview; EFOS d.o.o., Hruševje, Slovenia). The GST was 29 cm long × 19 cm wide × 20 cm tall with two 22.75‐cm^2^ triangular openings and was equipped with a removable sticky liner (330 cm^2^) coated with a hot‐melt pressure sensitive adhesive. Both ODT and GST traps were baited with a proprietary binary experimental lure (Trécé Inc.) consisting of a black PVC rectangle (2.7 cm × 2.4 cm) loaded with codlemone, pear ester, DMNT, and linalool oxide pyranoid in the same ratios reported previously.^13^, ^14^ The second lure was a white membrane cup (3.3 × 2.9 cm) loaded with acetic acid, which has been previously described.^26^ These two lures were placed next to each other in the middle of the sticky liner of both traps.

The GST consisted of a main body housing the electronic components powered by two lithium‐ion batteries (nominal voltage 3.7 V, rated capacity 2.2 Ah) and a solar panel (size 200 × 180 × 18 mm, power 4 W). The GST components included four high‐definition cameras (5 Mpixel), a GPRS modem (currently compliant with 2G, 3G, 4G, LTE and LTE‐M networks), a GPS, and a SIM card to send the capture pictures (and any other field data, including temperature and moisture collected by the weather sensor) to a proprietary cloud‐based IT platform. The high‐resolution images taken from the four cameras were stitched together by the software into a single picture on the screen. Each picture was stamped with location, day, and hour. The system power consumption allowed a maximum of three pictures per day and requires 100–200 MB of storage per month. In our study, the GST was programmed to take three pictures per day and the detection algorithm was set for codling moth.

### 2.2 Field experiments

Five orchards, including both apple (*Malus domestica* Borkhausen) and pear (*Pyrus communis* L.), were selected from across the Emilia‐Romagna region in Northern Italy to conduct four experiments in 2020 (see Table S1). Orchards with a history of limited chemical sprays applied over the previous 5 years were selected to create the opportunity to monitor codling moth among a more likely diverse pests assemblage. In each experiment, the position of five pairs of traps (GST and ODT) were randomized with traps placed at least 30 m from the orchard perimeter, 30 m apart, and in the upper part of the canopy, at 3 m.

Four sets of experiments were conducted during the 2020 growing season. Experiment #1 compared traps placed in the five orchards from 23–26 May to 18–26 June, during the first codling moth flight. Experiment #2 was conducted in the same five orchards from 18–26 June to 15–16 July, during the second codling moth flight. Experiments #3 and #4 compared all traps placed in a single apple orchard from 18 July to 29 July, during the second codling moth flight, and again from 29 July to 19 August, during the third codling moth flight, respectively.

Three pictures were taken daily by the smart trap in each experiment, at 20:00, 23:00, and 02:00 during the first two experiments and at 20:00, 00:00 and 05:00 in the last two experiments. These times were adjusted prior to the start of the study to reduce visual distortions in the picture images, i.e. dew on camera lenses in the morning hours and excessive light shadows in late afternoon. We manually validated pictures twice a week (every 3–4 days), observing the pictures from a computer monitor. Since the proprietary detection algorithm kept track of each moth's exact position on the sticky liner, liners were not replaced during each experiment.

Moth counts in the smart trap were assessed in two ways. First, each picture was visually inspected on a computer monitor to compare counts marked automatically by the detection algorithm with the numbers manually counted on the computer screen. Second, captures were verified by visiting traps directly in the field. Since data provided by the manual check of the traps were found to be consistent with the data obtained by manual inspection of the computer images, on‐screen counts performed by the human operator were considered reliable and were used for further data elaborations. Data were organized into four groups: automatic counts of codling moth, on‐screen manual counts of codling moth, false‐positive counts (automatically and incorrectly scored as codling moth), and false‐negative counts (codling moth adult captures which were missed by the detection algorithm). All false‐positive counts were subsequently identified manually as one of several nontarget arthropods or as one of several types of visual anomalies, including the shadow of the lure and aggregations of moth scales on the liner. In a few cases, the detection algorithm double‐counted a codling moth adult when the wings of the moth were expanded on the liner. In each trap, moths were also counted and sexed at the end of each experiment.

### 2.3 Statistical analyses

Statistical analyses were performed with R software version 4.0.2 (R Core Team 2020),^34^ including the packages lme4 and multcomp.^35^, ^36^ Figures were created using the package ggplot2.^37^ The effect of trap type on moth captures was analysed with a linear mixed model (lmer) from the lme4 package. Trap type and crop were considered as predictors, while the location in the model was a random effect. To test differences among GSTs, the data were scaled and analysed using a lmer. Trap ID and experiment were considered as predictors, while the automatic count and the counting error were added as controlling variables. A multiple comparison *post hoc* test was performed on the fitted model (glht function from multcomp package). The Pearson's correlation coefficient (*R* ^2^) between automatic and manual counts in the GSTs was calculated for the five smart traps and the four experiments using the ‘cor’ function. Four subsets of 50 pictures containing the major nontarget species were analysed with linear regression (lm). These major nontarget species counts, both the total present and the automatically miscounted on the sticky liner, were used as predictors to explain the false‐positive counts. Values in the text are reported as mean ± SEM.

## 3 RESULTS

**Figure 1:**
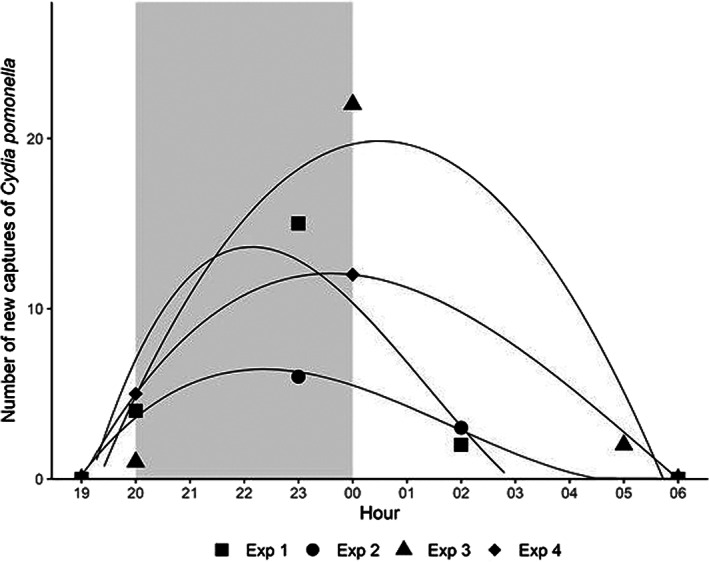
Composite of diurnal captures of *Cydia pomonella* adults in five smart traps taken at three time periods over four experiments conducted in Italy, 2020.

The cumulative counts of codling moth captured in the two traps (ODT and GST) over the entire study period were not significantly different (ODT 10.7 ± 1.7 *versus* GST 6.3 ± 1.1 adults per trap, *P* = 0.903). During experiment #1 the ODT captured 8.0 ± 2.9 codling moths and the GST captured 8.4 ± 2.7 codling moths, while in experiment #2 the ODT captured 4.0 ± 2.0 codling moths and the GST captured 3.0 ± 2.0 codling moths, in both cases without significant differences (*P* = 0.928 and *P* = 0.727, respectively). However, the ODT outperformed the GST in experiment #3 (16.8 ± 1.5 *versus* 9.4 ± 1.2, *P* = 0.005) and experiment #4 (13.8 ± 3.5 *versus* 4.2 ± 1.4, *P* = 0.034), both conducted in a single orchard later in the season. The proportions of female codling moths were also similar in both traps (ODT 0.38 ± 0.06 *versus* GST 0.37 ± 0.07, *P* = 0.887). The diurnal pattern of codling moth capture in the GST showed that the peak response to the five‐component lure occurred between 20:00 and 00:00 (Fig. 1).

**Figure 2:**
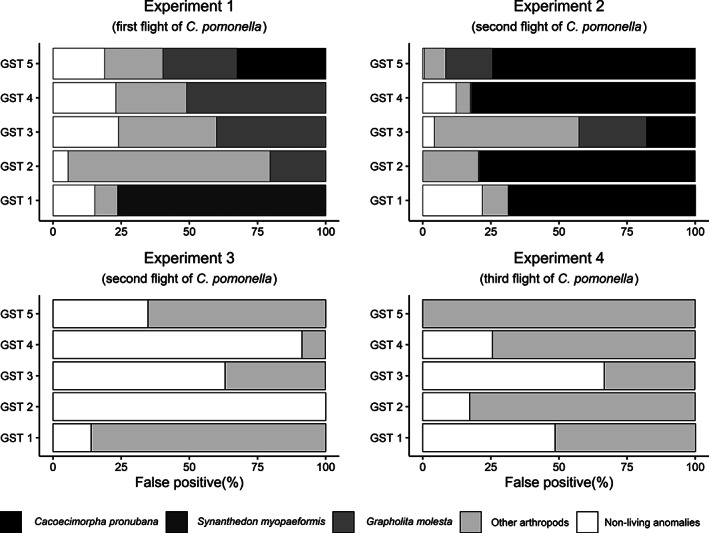
Major contributors to false‐positive counts of *Cydia pomonella* by the smart trap. Experiments #1 and #2 were conducted in five separate orchards and experiments #3 and #4 were both conducted in one location.

Three major moth pests plus muscid flies (Diptera: Muscidae) accounted for most of the nontarget arthropods captured in traps during experiments #1 and #2. These included oriental fruit moth, apple clearwing moth, and carnation tortrix, *Cacoecimorpha pronubana* (Hübner) (Lepidoptera: Tortricidae). The ODT and GST in both experiments captured similar numbers of these three nontarget pest species: oriental fruit moth (27 *versus* 24 individuals), apple clearwing moth (19 *versus* 12 individuals), and carnation tortrix (184 *versus* 102 individuals). However, the presence and abundance of these nontarget pest species varied among the tested orchards (Fig. 2). None of these three moth species were captured in the sole apple orchard used for the latter two experiments.

The correlation between automatic and manual counts in the GST varied according to the abundance and variability of nontarget captures in the different orchards (Table 1). When the smart traps captured codling moth together with an assemblage of nontarget species, the correlation was lower in traps with a higher number of false‐positive counts (R^2^ ranging from 0.66 ± 0.10 to 0.87 ± 0.03). Similarly, the percentage of false‐positive and ‐negative automatic identifications varied across orchards and experiments (Table 1). A comprehensive data analysis of all the pictures collected across the four experiments showed that both false‐positive and false‐negative counts were significantly different among traps (df = 4, 1253, *F* = 24.12, *P* < 0.001 and *F* = 21.12, *P* < 0.001, respectively). The same result was obtained analysing the single experiments (data not shown).

**Table 1:** *Cydia pomonella* identification errors in the smart traps over four field experiments conducted in Italy in 2020

	Mean values ± SEM per picture^a^			
Identification error	Experiment #			
	1	2	3	4
Automatic count of *C. pomonella*	8.5 ± 0.3	4.1 ± 0.2	6.2 ± 0.3	2.6 ± 0.1
Manual count of *C. pomonella*	6.6 ± 0.3	1.6 ± 0.1	5.5 ± 0.3	2.0 ± 0.1
Correlation coefficients (*R* ^2^)	0.75 ± 0.04	0.66 ± 0.10	0.87 ± 0.03	0.70 ± 0.12
False positive (%)	32.1 ± 1.4	66.8 ± 1.9	20.5 ± 2.3	36.8 ± 2.4
False negative (%)	5.4 ± 0.7	2.0 ± 0.9	3.4 ± 0.6	2.6 ± 0.6

The automatic counts were provided by the detection algorithm, while the manual counts corresponded to the visual observations of the operator. The Pearson's correlation coefficients (*R* ^2^) was calculated between automatic and manual counts.

^a^ In experiments #1 and #2, single smart traps were placed in five different pome fruit orchards, while in experiments #3 and #4 all five smart traps were compared in one location. Data analysis was performed on 384 pictures in experiment #1, 389 in experiment #2, 170 in experiment #3 and 315 in experiment #4.

The three nontarget insect pests (apple clearwing moth, oriental fruit moth, and carnation tortrix) that were of a relatively similar size as codling moth contributed the largest proportion of false‐positive identifications. Similarly, the detection algorithm misidentified captures of flies (Diptera: Muscidae) (body length 7–10 mm) and in limited cases other species with body sizes 5–15 mm, including noctuids (Lepidoptera: Noctuidae), ants (Hymenoptera: Formicidae), earwigs (Dermaptera: Forficulidae), honeybees (Hymenoptera: Apidae), grasshoppers (Orthoptera), and jumping spiders (Araneae: Salticidae). Various noninsect objects were occasionally scored as codling moth adults, such as deposits of moth scales, the lures placed on the liners, or their shadows. A small number of false‐positive counts occurred when single moths stuck on the liner with extended wings were scored as two adults (Fig. 2).

Within the subsample of pictures in which the four major nontarget species occurred most frequently (Table 2), it is noteworthy that only a part of the nontarget individuals present on the sticky liner were detected and counted as codling moth, impacting the percentage of false‐positive counts. In these subsets of data considered as ‘worst cases’, the number of apple clearwing moths miscounted as codling moth had a significant effect on the false‐positive counts (df = 2, 47, *F* = 46.21, *P* < 0.001), while the total apple clearwing moth individuals present on the sticky liners did not impact the false‐positive counts (df = 2, 47, *F* = 46.21, *P* = 0.539). Similarly, the number of carnation tortrix moths miscounted as codling moth had a significant effect on the false‐positive counts (df = 2, 47, *F* = 91.18, *P* < 0.001), while the total carnation tortrix individuals present on the sticky liners did not impact the false‐positive counts (df = 2, 47, *F* = 91.18, *P* = 0.437). On the contrary, in the case of oriental fruit moth, both the number of individuals automatically detected and the total number present on the sticky liners had a significant effect on false‐positive counts (df = 2, 46, *F* = 4.31, *P* = 0.027 and df = 2, 46, *F* = 4.31, *P* = 0.005, respectively). Likewise, both the total number of flies present on the sticky liners and the number of flies detected by the algorithm significantly impacted the false‐positive counts (df = 2, 32, *F* = 55.34, *P* < 0.001), demonstrating that for these latter species the majority of the individuals captured were miscounted by the detection algorithm and when present they largely contributed to the false‐positive counts.

**Table 2:** Occurrences of false‐positive identifications of *Cydia pomonella* in smart traps placed in orchards with the co‐occurrence of several major nontarget species: *Synanthedon myopaeformis*, *Grapholita molesta*, *Cacoecimorpha pronubana*, and flies (Diptera: Muscidae)

Analyses of smart trap pictures	Mean values ± SEM per picture^a^			
	Major nontarget			
	*S. myopaeformis*	*G. molesta*	*C. pronubana*	Flies
Automatic count of *C. pomonella*	8.3 ± 0.3	18.9 ± 0.5	7.1 ± 0.4	21.2 ± 0.4
Manual count of *C. pomonella*	6.6 ± 0.3	15.7 ± 0.3	0.0 ± 0.0	17.2 ± 0.1
All false positives counted as *C. pomonella*	1.9 ± 0.1	4.4 ± 0.3	7.1 ± 0.4	4.9 ± 0.4
Major non‐target counted as *C. pomonella*	1.6 ± 0.1	2.2 ± 0.2	5.2 ± 0.4	2.7 ± 0.3
Actual captures of major nontarget	6.2 ± 0.5	7.9 ± 0.2	26.7 ± 1.5	6.6 ± 0.5
Percentage of the major nontarget scored as false positives	86.0 ± 2.9	60.3 ± 5.0	72.2 ± 2.3	56.1 ± 4.4
Percentage of the major nontarget miscounted out of the total captured	37.0 ± 4.4	33.7 ± 4.1	20.6 ± 1.4	43.3 ± 4.0

^a^ *S. myopaeformis* counts were taken from a subset of 50 pictures from experiment #1 in an apple orchard located in Villa Prati (RA), *G. molesta* and flies counts were taken from a subset of 49 and 35 pictures, respectively, from experiment #1 in a pear orchard located in San Cesario Sul Panaro (MO), and *C. pronubana* counts were taken from a subset of 50 pictures from experiment #2 in a pear orchard located in Baricella (BO).

## 4 DISCUSSION AND CONCLUSION

Remote insect pest monitoring is becoming an important complementary option for field scouting to support timely crop protection decisions.^15^, ^16^ Pome orchards are often attacked by a suite of moth pests which can be monitored with traps and the relatively high cost of smart traps could be reduced if traps could monitor more than one of these pests simultaneously. Yet, the effectiveness of using such automated devices for multiple pest species has never been previously explored. The smart trap evaluated in our study proved to be operationally reliable, i.e. battery performance, data transfer, and high quality images (Figs S1–S3) were provided without malfunctions. The smart trap performed similarly to a standard delta‐shaped trap in capturing codling moth; however, its automatic analysis of the images was often poor due to nontarget misidentifications in presence of baits less selective than sex pheromone lures. Although the manual examinations of the remote images were successful in counting codling moth without visiting the orchards, the inflated automatic counts recorded by the smart trap due to false‐positive identifications if ignored could cause an over‐response of managers to actual pest densities.

This study was deliberately conducted across several biologically diverse Italian pome fruit orchards to demonstrate the potential and limitations of using a remote trap to monitor codling moth when a diverse assemblage of other pest and nonpest species can occur in traps. Our results showed that the current image detection algorithm used in this study was not effective in differentiating codling moth from a diverse group of similar‐sized objects on the sticky liner, providing false‐positive counts. The common occurrence of miscounting of several types of similar‐sized objects was exacerbated by the use of the five‐component sex pheromone‐kairomone lure, which proved not to be selective for codling moth. We presume it is much more likely that this smart trap if baited with only codlemone would have been a reliable and convenient tool to remotely monitor codling moth as has been shown in previous studies due to the high lure selectivity.^21^, ^22^, ^23^ Nevertheless, the low percentage of false‐negative identifications recorded in our study demonstrated that the smart trap algorithm was capable of automatically recognizing codling moth on liners littered with other conflicting objects.

Another current limitation with smart traps is their inability to automatically differentiate moth sexes. Codling moth females are only slightly larger than males, and the two sexes have no distinguishing morphological characters without requiring a physical manipulation of the moth to observe the genitalia and the underside of the forewings.^38^ It is also impossible to accurately sex moths from examining the screen images. The development of bisexual lures and the need to improve both action thresholds and phenology models based on female counts could be considered as serious limitations for the future adoption of smart traps.^39^, ^40^ In addition, it is unlikely that smart traps could be used to differentiate ‘wild’ from laboratory‐reared moths, which is critical in assessing sterile insect technology programs.^41^, ^42^

Smart traps can be useful tools in various research projects involving pest biology, such as characterizing the diurnal flight patterns of tortricid species.^43^ Pictures taken at different timings in our study confirmed the period of adult codling moth activity, which has been well‐studied previously.^44^, ^45^, ^46^ This ability to view trap liners at several times per day could be used to elaborate the influence of photoperiod, temperature, wind speed, and other environmental parameters on the behaviours of other important pests that have not been as thoroughly studied as codling moth.

The precision of the automatic counts for codling moth, among an assemblage of multiple species, varied among orchards primarily based on the abundance and variety of nontarget items. Adults of all three major nontarget moth species were of comparable sizes to codling moth adults (6–11 mm expressed as forewing length): 8–13 mm for apple clearwing moth, 5–7 mm for oriental fruit moth, and 6–12 mm for carnation tortrix.^47^ When present, they significantly impacted the false positives within the automatic counts. In addition, moths on liners assumed a variety of final shapes as they stuggled to escape the adhesive (Figs S1–S3). However, other causes of machine over‐counting codling moth in our study could probably be alleviated. First, lures should not have been placed on the liner, but instead suspended from the inside apex of the trap. Second, the frequency of moth scales triggering a positive count could be reduced with the use of an adhesive that could minimize moth trails on the liner.^33^ Third, the performance of the machine's identification algorithms could likely be improved by adjusting the saturation of the sticky liner background colour and narrowing the acceptable range of the automatic identification parameters.^48^, ^49^ However, accurate counting on liners with overlapping images due to crowding remains problematic.^50^ While weekly counts of codling moth are typically low, trap liners will need to be replaced throughout the season to avoid saturation and image integrity.^51^ Maintaining the quality of traps and liners, of course, requires visitation, and likely reduces the value added from remote trapping. In addition, other factors such as the trap colour can potentially affect both the captures of the target pest and the bycatches of other nontarget species, contributing to the saturation of the sticky liners and increasing the need for direct field visits, although it seems not to be the case for the colours used in our study.^52^, ^53^

The use of remote smart traps provides an opportunity to reduce pest monitoring costs and increase their use in time‐sensitive decision making.^23^ In addition, automated pest monitoring can be easily integrated into decision aids.^54^, ^55^ However, further improvements in target differentiation are needed to be able to monitor multiple tortricid species and to use effective bisexual lures which can improve decision making.^26^, ^56^ The adoption of such smart technologies is also dependent on its coupling with trained field staff.^16^

## Conflict of Interest

The authors declare no competing interests. The mention of trade names or commercial products in this publication is solely to provide specific information and does not imply recommendation or endorsement by the author institutions.

## Supporting information

**Table S1.** Four *Cydia pomonella* remote monitoring experiments were performed in 2020 in Italy with a camera‐equipped smart trap referred to as a green smart trap (GST) in comparison with the standard delta‐shaped trap referred to as an orange delta‐shaped trap (ODT). The selected pome fruit orchards were either unsprayed or managed according to organic farming with the use of mating disruption (MD). Experiments #1 and #2 were conducted comparing one pair of a GST and an ODT in five locations, while experiments #3 and #4 were subsequently conducted with five pairs of a GST and an ODT in one location.
**Figure S1.** Picture from a smart trap taken on 31 May at 23:00 in an apple orchard located in Villa Prati (Ravenna, Italy) during experiment #1.The picture shows two monitoring lures (a black PVC dispenser and a white membrane cup), eight *Cydia pomonella* individuals marked by green squares, and three unmarked *Synanthedon myopaeformis* individuals.
**Figure S2.** Picture from smart trap taken on 31 May at 23:00 in a pear orchard located in San Cesario Sul Panaro (Modena, Italy) during experiment #1. The picture shows two monitoring lures (a black PVC dispenser and a white membrane cup), 15 *Cydia pomonella* individuals marked by green squares, and eight unmarked *Grapholita molesta* individuals.
**Figure S3.** Picture from a smart trap taken on 1 July at 23:00 in a pear orchard located in Baricella (Bologna, Italy) during experiment #2. The picture shows two monitoring lures (a black PVC dispenser and a white membrane cup), 13 *Cacoecimorpha pronubana* individuals representing false positive counts marked by yellow squares, and several other nontargets unmarked, including *C. pronubana* and moth scales.
