# Supplementary material for: Remote monitoring of Cydia pomonella adults among an assemblage of nontargets in sex pheromone‐kairomone‐baited smart traps
Source: Pest Manag Sci. 2021 May 14;77(9):4084–90. doi: 10.1002/ps.6433 (PMC8453955; doi:10.1002/ps.6433)
Supplement: Supplementary file 1 — Table S1. Four Cydia pomonella remote monitoring experiments were performed in 2020 in Italy with a camera‐equipped smart trap referred to as a green smart trap (GST) in comparison with the standard delta‐shaped trap referred to as an orange delta‐shaped trap (ODT). The selected pome fruit orchards were either unsprayed or managed according to organic farming with the use of mating disruption (MD). Experiments #1 and #2 were conducted comparing one pair of a GST and an ODT in five locations, while experiments #3 and #4 were subsequently conducted with five pairs of a GST and an ODT in one location. Figure S1. Picture from a smart trap taken on 31 May at 23:00 in an apple orchard located in Villa Prati (Ravenna, Italy) during experiment #1.The picture shows two monitoring lures (a black PVC dispenser and a white membrane cup), eight Cydia pomonella individuals marked by green squares, and three unmarked Synanthedon myopaeformis individuals. Figure S2. Picture from smart trap taken on 31 May at 23:00 in a pear orchard located in San Cesario Sul Panaro (Modena, Italy) during experiment #1. The picture shows two monitoring lures (a black PVC dispenser and a white membrane cup), 15 Cydia pomonella individuals marked by green squares, and eight unmarked Grapholita molesta individuals. Figure S3. Picture from a smart trap taken on 1 July at 23:00 in a pear orchard located in Baricella (Bologna, Italy) during experiment #2. The picture shows two monitoring lures (a black PVC dispenser and a white membrane cup), 13 Cacoecimorpha pronubana individuals representing false positive counts marked by yellow squares, and several other nontargets unmarked, including C. pronubana and moth scales. [file PS-77-4084-s001.docx]

# Supplemental material

**Table S1**. Four *Cydia pomonella* remote monitoring experiments were performed in 2020 in Italy with a camera-equipped smart trap referred as GST (green smart trap) in comparison with a standard delta-shaped trap referred as ODT (orange delta trap). The selected pome fruit orchards were either unsprayed or managed according to organic farming with the use of mating disruption (MD). Experiments #1 and #2 were conducted comparing one pair of GST and ODT in five locations, while experiments #3 and #4 were subsequently conducted with five pairs of GST and ODT in one location.

| Exp.  # | Location  (province) | Geocoordinates | Crop, cultivar and block details (training system, planting year and planting space) | Block size | Grower  Spray program |
| --- | --- | --- | --- | --- | --- |
|  |  |  |  |  |  |
| 1, 2, 3, 4 | Villa Prati  (RA) | 44°27'33.65"N  12° 1'43.48"E | Apple cv Gold Rush, spindel, 2013,  4 m × 1 m | 3.0 ha | Organic with MD |
|  |  |  |  |  |  |
| 1, 2 | Renazzo  (FE) | 44°46'12.93"N  11°18'8.00"E | Pear and Apple cv Abate Fetel and Early Gala, spindel, 2013, 4 m × 1 m | 1.2 ha | Organic  with MD |
|  |  |  |  |  |  |
| 1, 2 | Dosso  (FE) | 44°46'4.07"N  11°19'34.55"E | Apple cv Mele del Privale (Gala group), spindel, 2015, 4 m × 1 m | 1.2 ha | Organic with MD |
|  |  |  |  |  |  |
| 1, 2 | San Cesario sul Panaro (MO) | 44°34'2.39"N  11° 0'46.83"E | Pear cv Abate Fetel and Bartlett, palmette, 2004, 4 m × 2.5 m | 0.5 ha | Unsprayed |
|  |  |  |  |  |  |
| 1, 2 | Baricella  (BO) | 44°38'54.28"N  11°32'41.17"E | Pear cv Abate Fetel and Decana, spindel, 1990, 4 m × 1 m | 0.4 ha | Unsprayed |
|  |  |  |  |  |  |

During exp. #1 and #2 the five camera-equipped traps were assigned to the following locations: GST 1 was placed in Villa Prati, GST 2 in Renazzo, GST 3 in San Cesario sul Panaro, GST 4 in Dosso and GST 5 in Baricella. During exp. #3 and #4, all five traps were deployed in Villa Prati.


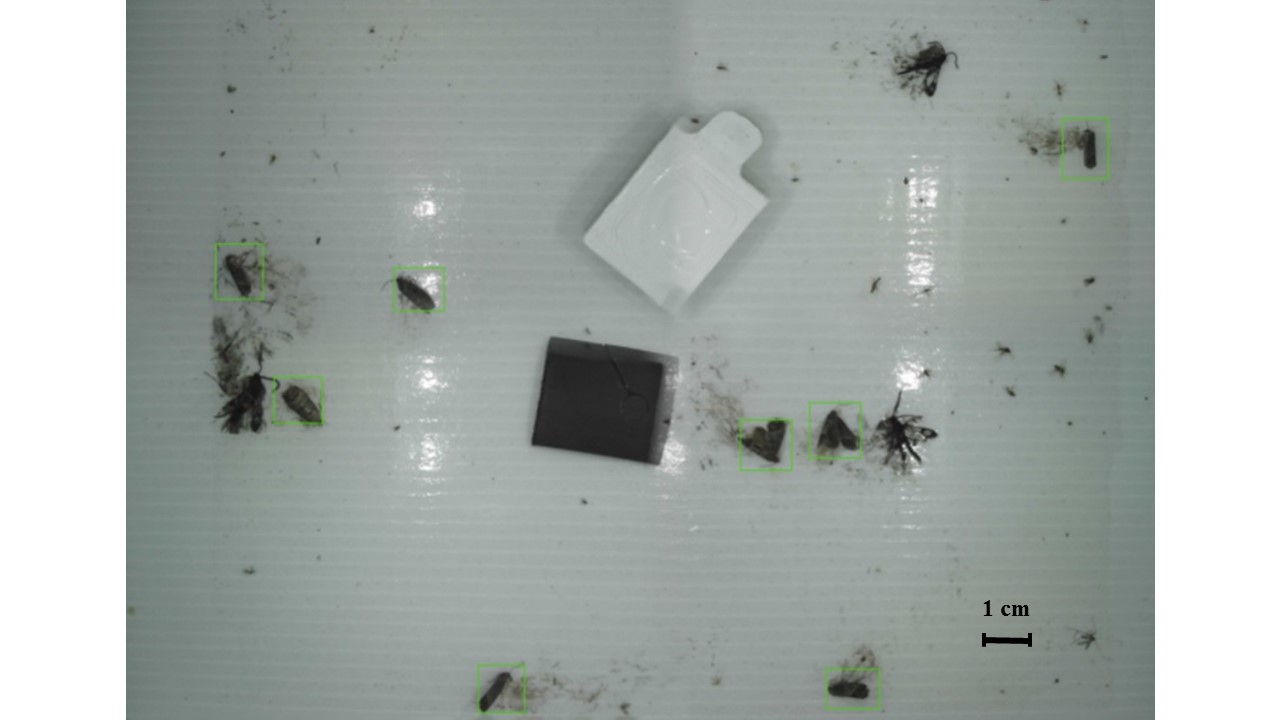


**Figure S1.** Picture from smart trap taken on 31 May at 23:00 in an apple orchard located in Villa Prati (Ravenna, Italy) during experiment #1.The picture shows two monitoring lures (a black PVC dispenser and a white membrane cup), eight *Cydia pomonella* individuals marked by green squares, and three unmarked *Synanthedon myopaeformis* individuals.


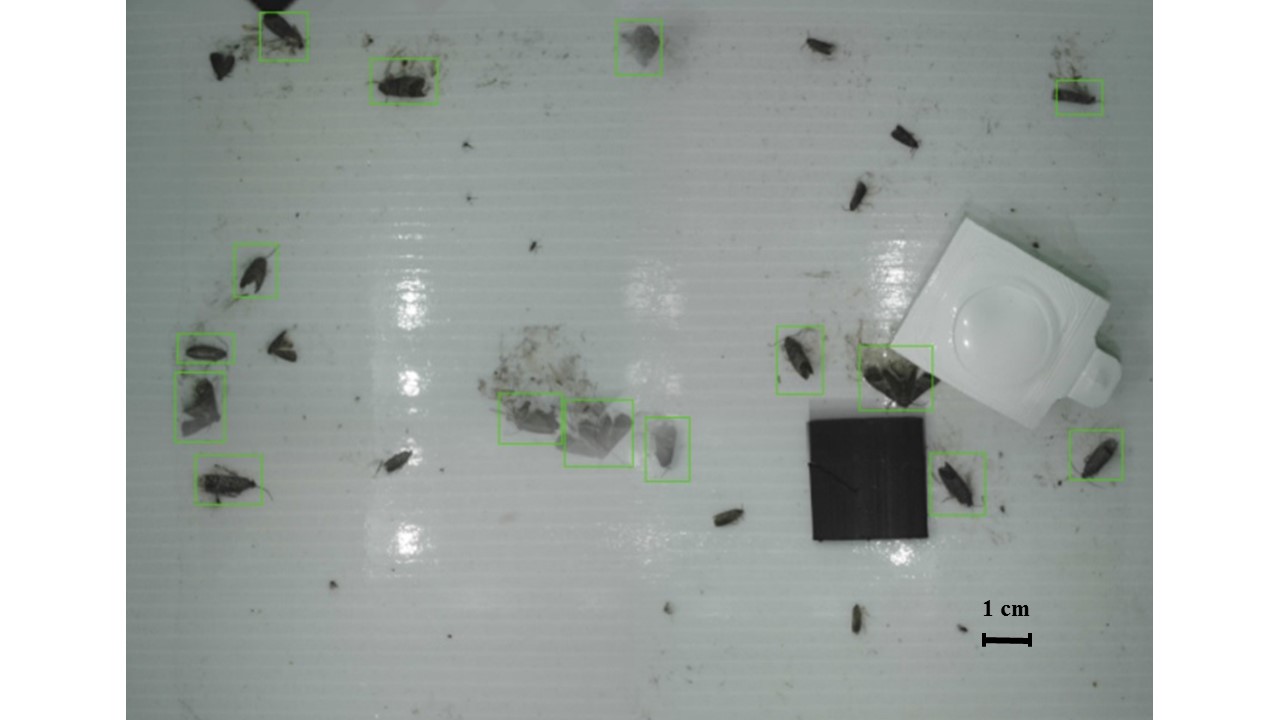


**Figure S2.** Picture from smart trap taken on 31 May at 23:00 in a pear orchard located in San Cesario Sul Panaro (Modena, Italy) during experiment #1. The picture shows two monitoring lures (a black PVC dispenser and a white membrane cup), fifteen *Cydia pomonella* individuals marked by green squares, and eight unmarked *Grapholita molesta* individuals.


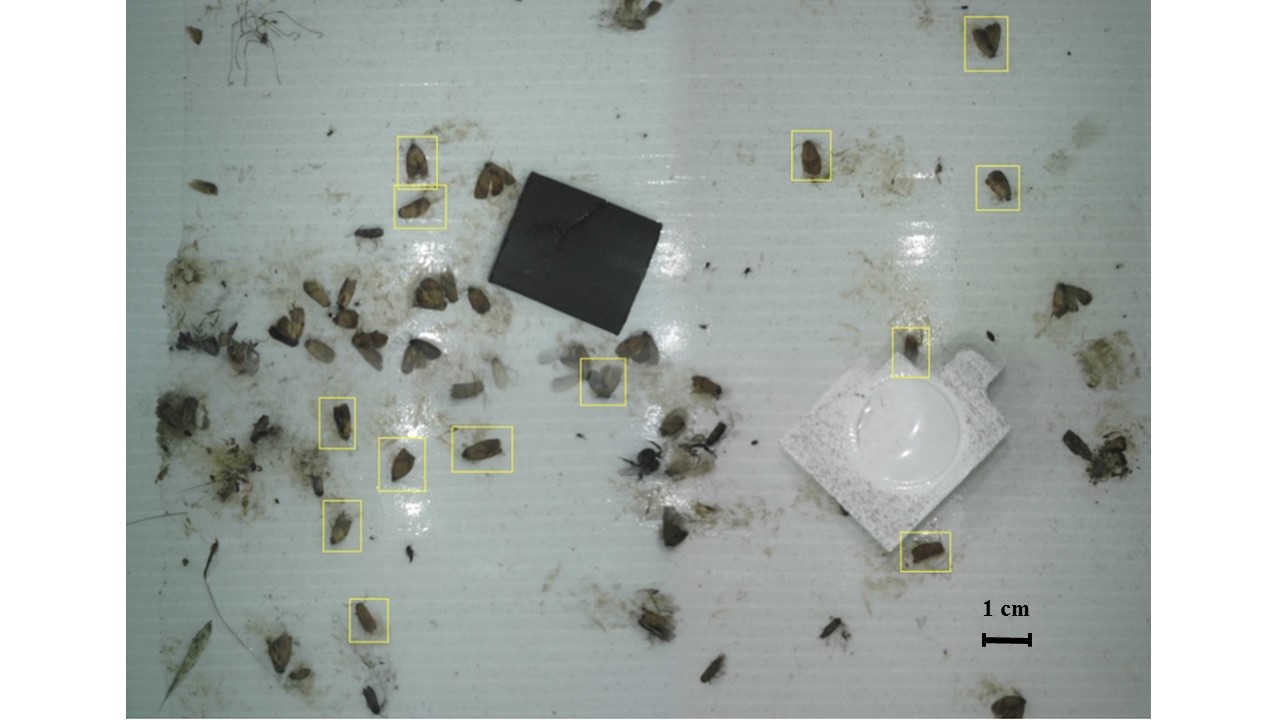


**Figure S3.** Picture from smart trap taken on 1 July at 23:00 in a pear orchard located in Baricella (Bologna, Italy) during experiment #2. The picture shows two monitoring lures (a black PVC dispenser and a white membrane cup), thirteen *Cacoecimorpha pronubana* individuals representing false positive counts marked by yellow squares and several other non-targets unmarked, including *C. pronubana* and moth scales.
